# ceRNA Network Regulation of TGF-β, WNT, FOXO, Hedgehog Pathways in the Pharynx of *Ciona robusta*

**DOI:** 10.3390/ijms22073497

**Published:** 2021-03-28

**Authors:** Aiti Vizzini, Angela Bonura, Laura La Paglia, Antonino Fiannaca, Massimo La Rosa, Alfonso Urso, Vincenzo Arizza

**Affiliations:** 1 Dipartimento di Scienze e Tecnologie Biologiche, Chimiche e Farmaceutiche–Università di Palermo, via Archirafi 18, 90100 Palermo, Italy; vincenzo.arizza@unipa.it; 2 Istituto per La Ricerca e l’Innovazione Biomedica–Consiglio Nazionale Delle Ricerche, via Ugo La Malfa 153, 90100 Palermo, Italy; angela.bonura@irib.cnr.it; 3 Istituto di Calcolo e Reti ad Alte Prestazioni–Consiglio Nazionale Delle Ricerche, via Ugo La Malfa 153, 90100 Palermo, Italy; laura.lapaglia@icar.cnr.it (L.L.P.); antonino.fiannaca@icar.cnr.it (A.F.); massimo.larosa@icar.cnr.it (M.L.R.); alfonso.urso@icar.cnr.it (A.U.)

**Keywords:** NGS, TGF-β, WNT, FOXO, miRNA, pseudogenes, ascidian

## Abstract

The transforming growth factor-β (TGF-β) family of cytokines performs a multifunctional signaling, which is integrated and coordinated in a signaling network that involves other pathways, such as Wintless, Forkhead box-O (FOXO) and Hedgehog and regulates pivotal functions related to cell fate in all tissues. In the hematopoietic system, TGF-β signaling controls a wide spectrum of biological processes, from immune system homeostasis to the quiescence and self-renewal of hematopoietic stem cells (HSCs). Recently an important role in post-transcription regulation has been attributed to two type of ncRNAs: microRNAs and pseudogenes. *Ciona robusta*, due to its philogenetic position close to vertebrates, is an excellent model to investigate mechanisms of post-transcriptional regulation evolutionarily highly conserved in immune homeostasis. The combined use of NGS and bioinformatic analyses suggests that in the pharynx, the hematopoietic organ of *Ciona robusta*, the Tgf-β, Wnt, Hedgehog and FoxO pathways are involved in tissue homeostasis, as they are in human. Furthermore, ceRNA network interactions and 3′UTR elements analyses of Tgf-β, Wnt, Hedgehog and FoxO pathways genes suggest that different miRNAs conserved (cin-let-7d, cin-mir-92c, cin-mir-153), species-specific (cin-mir-4187, cin-mir-4011a, cin-mir-4056, cin-mir-4150, cin-mir-4189, cin-mir-4053, cin-mir-4016, cin-mir-4075), pseudogenes (ENSCING00000011392, ENSCING00000018651, ENSCING00000007698) and mRNA 3′UTR elements are involved in post-transcriptional regulation in an integrated way in *C. robusta*.

## 1. Introduction

The transforming growth factor-β (TGF-β) is a cytokine involved in regulation of cell fate and behavior in all tissues of the body [1]. In human hematopoiesis, TGF-β plays an important role in regulating hematopoietic stem cell (HSC) behavior, particularly in quiescence and self-renewal [2]. TGF-β signals are activated through a canonical small mother against decapentaplegic (SMAD)-mediated) pathway. Following the binding with the ligand, type II receptors and type I receptors trigger transmit intracellular signaling through the phosphorylation of downstream effector SMADSs [3,4,5]. The SMAD pathway is fully integrated into the intracellular signaling network, to address the expression and activities of ligands, antagonists, receptors by interactions with intracellular signaling such as Wintless (WNT), Hedgehog (HH), phosphoinositide 3-kinase (PI3K)-Akt [6], nuclear factor kB (NF-kB), and Janus kinases (JAKs), signal transducer and activator of transcription proteins (STATs) signaling pathways [7,8].

Recent evidence indicates an association between different noncoding RNAs (ncRNAs) and TGF-β signaling [9]. An important role in the post-transcriptional regulation of genes is attributed to two type of ncRNAs: microRNAs (miRNAs) and pseudogenes. miRNAs interact in a regulative network as competitive endogenous RNA molecules (ceRNAs), driving the transcription of the target RNA messenger (mRNA). miRNAs are ∼22-nucleotide (nt) ncRNAs that regulate the biological processes through recognition of target elements of nucleotides 2–7 long in 3′ untranslated regions (3-UTRs) of mRNA target [10]. The result of miRNA targeting is typically a modest reduction in protein output [11] by deadenylation and subsequent mRNA degradation [12,13,14]. miRNA may exert an impact on cell fate by targeting multiple components of regulatory networks and this has been demonstrated experimentally, by measuring mRNA abundance following miRNA perturbations [15]; bioinformatically, by using multiple features of target sites and pathway annotations [16]; and by screening for direct miRNA targets [17]. Pseudogenes have been identified from prokaryotes to eukaryotes [18] and for a long time have been considered to be “junk DNA”, “genomic fossils” and “gene relics” [19,20]. Next-generation sequencing (NGS) technology has amply showed that pseudogenes are involved in transcriptional and posttranscriptional modulation of gene expression, and also in the maintenance of homeostasis. A fraction of pseudogenes has a protein-coding capacity [21], suggesting that can act as elements that contribute to the proteome.

The urochordate *Ciona robusta*, that shows a close phylogenetic relationship with vertebrates [22,23,24,25], is a powerful animal model in various fields of biology, serving for studying topics ranging from developmental biology to immunity in comparative study [26]. Recently, it has become an excellent model for the study of the evolution of post-transcriptional regulatory mechanisms in genes expression. In immunity response, multiple genes, within distinct functional categories, are coordinately and temporally regulated by transcriptional ‘on’ and ‘off’ switches which respond to external stimuli as lipopolysaccharides (LPS) and mechanisms that control transcriptional and post-transcriptional regulation. This sequence of events is important in coordinating the initiation and resolution of inflammation. The pharynx of *C. robusta* is the main haemopoietic organ, and can be challenged by inoculating LPS and can express immune related genes such as mannose binding lectin-like (*Mbl-like*) [27], galectin-like (*Gal-like*) [28], interleukins 17 (*Il-17*) [29] tumor growth factor β (*Tgf-β*) [30] and macrophage migration inhibitory factors (*Mif1* and *Mif2*) [31]. In human MIF is an important cytokine that has received substantial attention as a mediator of innate and adaptive immune response [32]. In *C. robusta Mif2* show cis-acting gamma interferon inhibitor of translation element (GAIT) and a cytoplasmic polyadenylation element (CPE) in 3′-UTR, which is not present in the 3′-UTR of *Mif1*, suggesting the presence of both transcriptional and post-transcriptional control mechanisms involved in the gene expression of the *Mif* genes (*Mif1*, *Mif2*) in innate immune response [31]. Furthermore, in C. robusta the presence of cis-acting GAIT elements in the 3′-UTR of *Tlr2*, *MyD88*, *IκB*, *Ikkα*, and *Nf-κB* mRNA confirmed that mechanisms involved in post-transcriptional regulation control of immune genes in human resulted highly conserved [33]. In *C. robusta* the signaling pathways and their post-transcriptional regulation in hematopoiesis are poorly studied and only some authors have described haematopoietic nodules in the pharynx in proliferation zones. In haemolymph, Lymphocyte-like cells (LLCs), can be marked by anti-CD34 monoclonal antibodies in agreement with the potential role of a pluripotent cell able to differentiate in different cell types [34].

RNA-Seq is becoming increasingly utilized to profile the annotated transcriptome, to detect and quantify known and novel transcripts, and identify novel classes of RNA. In the present study, using NGS and bioinformatics analysis we provide the first view of the pathways involved in homeostasis in the pharynx. Gene Ontology (GO) and Pathway analysis and ceRNA interaction analysis (miRNA-mRNA target-pseudogene) suggest that in *C. robusta* as in humans, the Tgf-β, Wnt, FoxO, and Hedgehog pathways could be involved in tissue homeostasis and that miRNAs, pseudogenes and elements of 3′-UTR are involved in post-transcriptional regulation of genes mechanisms that have been highly conserved during the evolution of animal species.

## 2. Results

### 2.1. Transcriptomic Analysis of Ciona Robusta Haemopoietic Organ

To profile *C. robusta* immunocompetent organ (pharynx) under physiological conditions we used next-generation sequencing (NGS) analyses

NGS identified 15,074 transcripts and 6729 of these were annotated by the Ensembl database (ensembl.org, release August 2021; NGS data shown in Supplementary Material files, transcriptome sheet). Of these transcripts, 99% were protein coding and the remaining 1% (201 elements) was classified as noncoding RNA (ncRNA). The 201 ncRNAs were divided as follows: 90 miRNAs, 27 pseudogenes, 19 small nuclear RNA (snRNAs), 36 small nucleolar RNAs (snoRNAs), 11 ribosomal RNAs (rRNAs), 10 miscellaneous RNAs (miscRNAs), and eight mitochondrial RNAs (mtRNAs) (Supplementary Material files, ncRNA annotation ensemble sheet).

**Figure 1:**
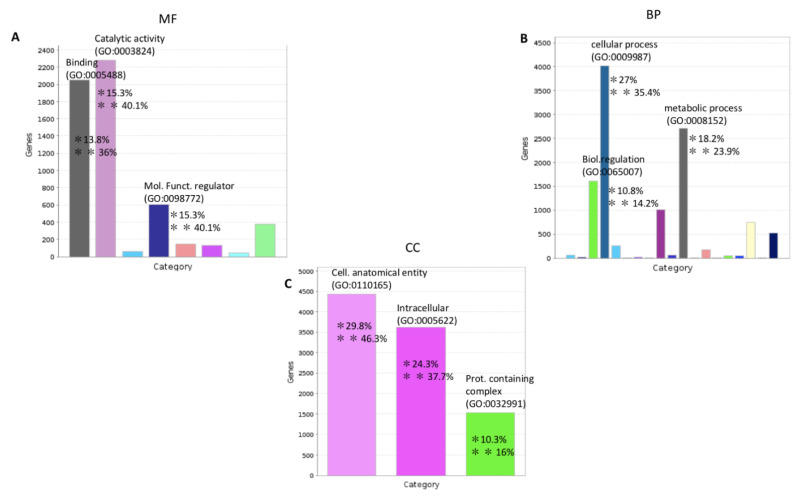
Functional classification with terms from Gene Ontology (GO) of the protein coding transcripts of *C. robusta*. The three GO classes are represented: (**A**): molecular function (MF), (**B**): biological process (BP), and (**C**): cellular component (CC). */** Chart tooltips are read as: category name (accession); * percent of gene hit against total # genes; ** percent of gene hit against total # function hits. Information is reported just for the three most representative subclasses for each GO class.

National Center of Biotechnology Information (ncbi) data (ncbi.nlm.nih.gov/genome/annotation_euk/Ciona_intestinalis/102; release October 2014) shows that 6% of transcripts in *C. robusta* genome are ncRNAs, including also RNA transfer (tRNAs) and long noncoding RNAs (lncRNAs). The different percentage of ncRNAs in our study (1% compared to 6%) is probably justified by the fact that we investigated only one organ (pharynx) and not the whole *C. robusta* transcriptome. Coding RNAs from NGS sequencing were analyzed through the functional classification system of a Gene Ontology (GO) tool (panther.org) (PANTHER 16.0, release April 2020), Supplementary Material, functional classification MF, BP and CC sheets (Figure 1).

**Figure 2:**
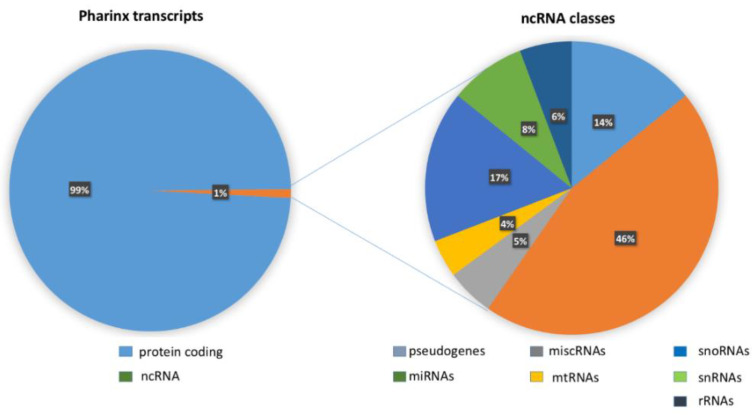
The left part of figure shows the whole pharynx transcripts of *C. robusta*. One percent of RNAs are noncoding. The right part of the figure shows the different classes of 1% of noncoding transcripts.

The GO analysis showed that the most representative categories for the molecular function subclass were: catalytic activity (GO:0003824), binding (GO:0005488), and molecular function regulator (GO:0098772). The most represented categories for the biological process subclass were: cellular process (GO:0048511), metabolic process (GO:0008152), and biological regulation (GO:0065007). For the cellular component subclass the most representative categories were: cellular anatomical entity (GO:0110165) and intracellular (GO:0005622). As shown in Figure 2, miRNAs and pseudogenes are the most representative classes of noncoding transcripts.

### 2.2. miRNAs Analysis

Of the 90 miRNAs identified by NGS, 54 were annotated and divided into two classes: 11 conserved miRNAs (Table 1) and 43 (Table 2) not conserved through the species (Supplementary Material, miRNA list sheet). Thirty-six of the 90 miRNAs were not annotated in the Ensembl database. Since the NGS sequences identified miRNA precursors (sequences from 59 to 77 base pairs long), we decided to align them using the miRBase Blastn tool (mirbase.org, release October 2018) to find a match with annotated mature miRNA sequences within the precursors (analysis results are in Supplementary Material, mirbase precursor and mature sheet). Only Blast results reporting an E-value < e − 5 = (10 − 5) were considered significant results and five potential mature miRNAs were identified: cin-mir-4105-5p, cin-mir-4198-5p, cin-mir-5596a-3p, cin-mir-5598-3p, cin-mir-5606-5p. Although just one miRNA sequence has an E-value score < e − 5 (cin-miR-5598-3p) and all precursor sequences identified with Blast research had E-values < e − 7; this difference is explained by the different length of sequence pairing when aligning precursor query sequences with mature miRNAs. A pipeline used to find miRNA annotations is shown in Supplementary Material (Figure S1).

To study the evolution pattern of the 11 conserved *C. robu*sta miRNA homologues in animal genomes (Table 3), we used the ZooMir (Supplementary Material ZooMir sheet) (insr.ibms.sinica.edu.tw/ZooMir/index.php, release April 2019), and MirGeneDB 2.0 (https://mirgenedb.org/, release 2015) database of homologous miRNA genes to search animal genomes that have been validated and annotated. Although cin-mir-375 is not present in the ZooMir list and cin-mir-141 is not present in MirGeneDB, both are present in miRBase database (http://www.mirbase.org/, release October 2018). The cin-mir-92c-5p, cin-mir-92a-3p, cin-mir-375-3p, cin-mir-153-5p and cin-let-7d-5p were highly conserved in chordate species (Table 3). miR-375 was highly conserved from *C. elegans* to *H. sapiens*, with one isoform except for *D. rerio* where there are two. Moreover, the miR-92 was conserved from *D. melanogaster* to *H. sapiens* and miR-let7 from *C. elegans* to *H. sapiens*.

**Figure 3:**
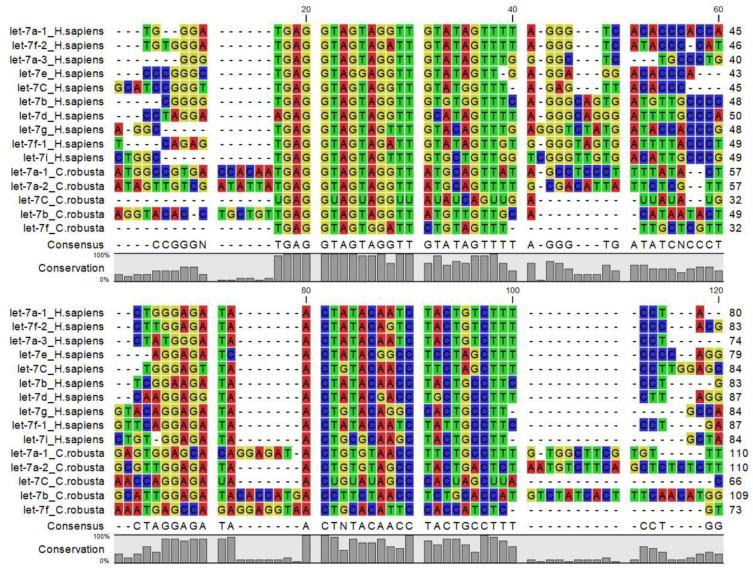
Multiple amino acid sequence alignment of Let7 miRNA family members from invertebrate, vertebrates and *C. robusta*.

The let-7 miRNA was one of the first miRNAs discovered in *C. elegans*, and results high conserved from nematode to the human. According to miRBase, *C. elegans*, *D. melanogaster*, *S. purpuratus*, *C. robusta*, *B. floridae*, *X. tropicalis*, *D. rerio*, G. *gallus*, *M. musculus* and *H. sapiens*, all express a version of let-7 (Table 3). let-7 miRNA orthologues share a consensus sequence called the ‘seed sequence’ GAGGUAG (Figure 3), that spans from nucleotides 2 through 8 of mature miRNA (data not shown), suggesting that their targets and functions may be similar in diverse animal species. The *C. elegans* and *D. melanogaster* have a single isoform, whereas chordates including *C. robusta* (mir-let-7a, a2, b, c, d, f) have multiple let-7 isoforms. Indeed *D. rerio* and *H. sapiens* have diverse let-7 family members (let-7a, -7b, -7c, -7d, -7e, -7f, -7g, -7h, -7i, -7j, -7k) although some differences may be observed (for example, let-7h exists in *D. rerio* but not in *H. sapiens*). According to miRBase, in human the let-7 family is composed of nine mature let-7 miRNAs encoded by 12 different genomic loci, some of which are clustered together (data not shown), in *D. rerio* the let-7 family is composed of eighteen mature let-7 miRNAs.

### 2.3. Pseudogene Characterization and Analysis

NGS technologies identified 27 pseudogenes in *C. robusta* that resulted annotated in Ensembl database and that were analyzed through Basic Local Alignment Search Tool (Blastn tool) (blast.ncbi.nlm.nih.gov/Blast.cgi, release October 2020), to find their parental gene and percentage of identity with parental genes (paralogues) (Table 4) (Supplemetary Material, pseudogene sheet). Pseudogenes showed a high percentage of identity with paralogue genes (94–100%) and some pseudogenes only had a few nucleotides that differed in sequence from their parental genes. Eleven of 27 pseudogene sequences were “predicted” by the Blast prediction algorithm, and the remaining 16 sequences were “validated”. All of them resulted in the same chromosome of parental genes and had lost their ability to encode proteins.

### 2.4. RNA-RNA Predictions

To investigate ceRNA interactions and possible involvement in the post-transcriptional regulation of pathway involved in pharynx tissue homeostasis, we tested all the transcripts produced with NGS technology, using the miRNATIP prediction tool.

**Figure 4:**
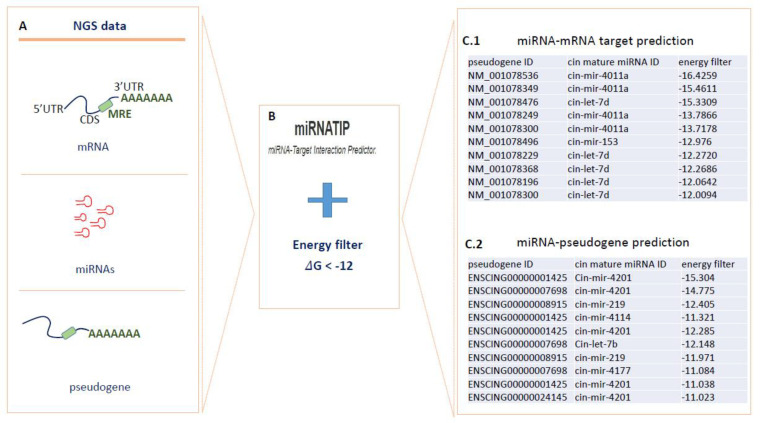
Prediction of mRNA–miRNA and miRNA–pseudogene interactions through the miRNATip algorithm. In (**A**–**C**), (**C.1**,**C.2**) are represented respectively the steps of the used pipeline: (**A**) different transcripts produced by NGS were analyzed by miRNATIP predictor. (**B**) miRNATIP computes interactions of a couple of RNA molecules (miRNA–mRNA and miRNA–pseudogenes). The predictions of all interactions are then filtered by the user through an energy filter of <−12 of ΔG. (**C.1**) miRNA–mRNA target prediction results filtered for energy values. (**C.2**) miRNA–pseudogenes prediction results filtered for energy values.

To consider, the most significant interactions, we filtered obtained results according to the lowest free energy values. All data of both interaction types are shown in Supplementary Materials. Figure 4 showed the RNA-RNA prediction pipeline. For miRNA-mRNA prediction, miRNATIP tool evidenced 341 interactions (228 unique genes with 20 different miRNAs), and five miRNAs were conserved through evolution (cin-mir-92c-5p, cin-mir-92a-3p, cin-mir-375-3p, cin-mir-153-5p and cin-let-7d-5p). miRNA-pseudogenes prediction evidenced 84 predictions (23 different pseudogenes with 50 different miRNAs). All miRNA-target interactions and miRNA-pseudogene interactions are in Supplemental Material (Supplementary Material miRNA-target prediction filter sheet and miRNA-pseudogene prediction sheet). These interactions that considered all annotated coding and noncoding transcripts had free energy < of −12 kcal/mol.

### 2.5. GO and Pathway Analysis

To investigate Gene Ontology (GO) annotations of targets which were predicted to interact with specific miRNAs and pseudogenes obtained by NGS sequences, gene enrichment and pathway analyses were performed using two different web servers: the Database for Annotation, Visualization and Integrated Discovery (DAVID) tool (david.ncifcrf.gov, release October 2016) and the Protein Analysis Through Evolutionary Relationships (PANTHER) tool (pantherdb.org, release April 2020).

**Figure 5:**
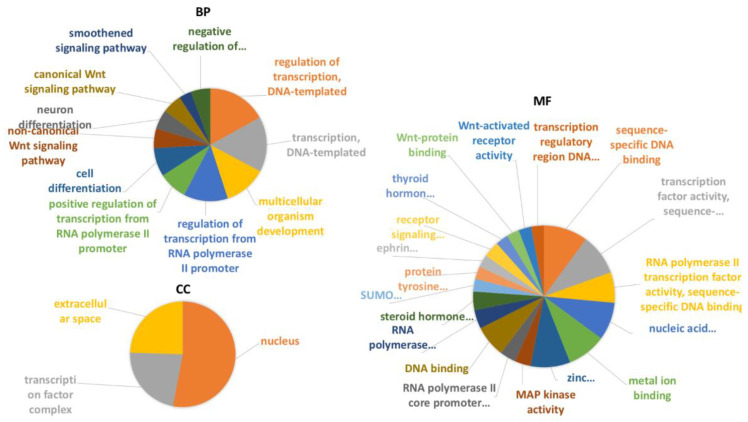
Gene Ontology enrichment analysis of *C. robusta* transcripts which were predicted to interact with specific miRNAs and pseudogenes obtained by NGS sequences. All the three different Gene Ontology subcategories were investigated: (i) biological pathways (BP); (ii) molecular functions (MF); (iii) and cellular components (CC).

Gene enrichment analysis was performed according to the three GO annotation classes: biological processes (BP), molecular functions (MF), and cellular component (CC) (all complete GO and pathway analysis are in Supplementary Material, BP, MF, CC, pathway kegg sheet). All GO results were filtered for the *p*-value statistical test (*p*-value < 0.05) and the Benjamini correction test (Benjamini < 0.05). Most representative terms were linked to WNT signaling (canonical and noncanonical pathway), MAP kinase activity, transcription regulation and protein binding. Figure 5 shows GO analysis of both BP, MF and CC classes. All GO results were filtered for *p*-value statistical test (*p*-value < 0.05) and Benjamini correction test (Benjamini < 0.05). Pathway analysis evidenced four pathways linked to homeostasis: the Tgf-β, Wnt, FoxO, and Hh pathways (Supplementary Material kegg pathway, Wnt gene list, FoxO gene list, Tgfβ gene list, Hh gene list sheets). Pathway results were filtered for the *p*-value statistical test (*p*-value < 0.05) and the Benjamini correction test (Benjamini < 0.05). Pathway selection was done filtering for the highest correction values of pathway analysis.

See Table 5 for a summary of pathway analyses and ceRNA interactions in selected pathways. (All GO and pathway analyses are shown in Supplementary Material, BP, MF, CC, kegg pathway sheet).

### 2.6. Identification of Structural Elements in the mRNA 3′-UTR of Tgf-β, Wnt, Hedgehog and FoxO Pathways Genes

**Figure 6:**
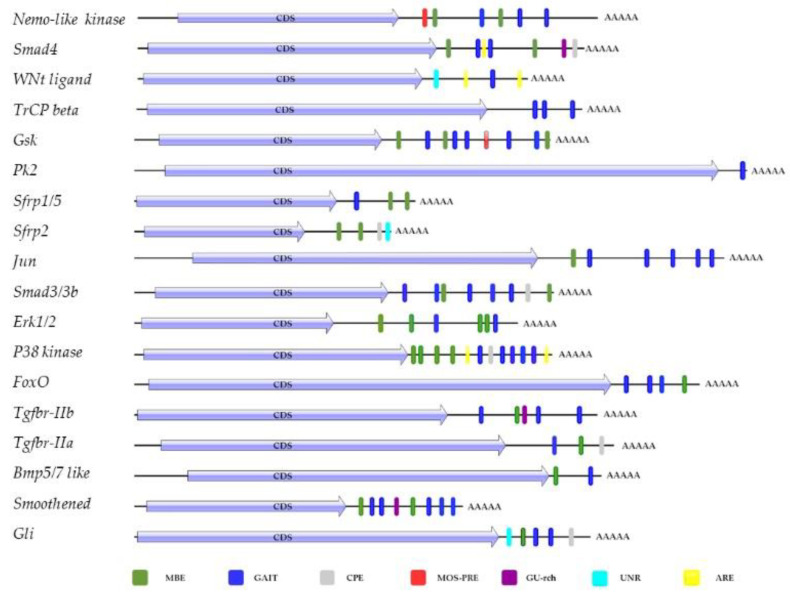
Analysis of 3′-UTR mRNA using the RegRNA web tool: MBE (musashi-binding element), GAIT (interferon-γ-activated inhibitor of translation), cytoplasmic polyadenylated element (CPE), MOS-PRE (polyadenylation response element), GU-rich destabilization element, RNA-binding protein (UNR), ARE (AU-rich element).

In silico analyses were performed using the RegRNA 2.0 tool (http://regrna2.mbc.nctu.edu.tw/ release February 2021) to investigate the cis-elements of the 3′-UTR involved in post-transcriptional regulation of *Tgf-β*, *Wnt*, *Hedgehog* and *FoxO* pathways genes (Figure 6).

Almost all mRNAs showed musashi-binding elements (MBE), and a γ-interferon activated inhibitor of translation (GAIT) element. In addition, RegRNA 2.0 identified in 3′-UTR of *Smad4*, *Smad2/3b*, *secreted frizzled-related protein* (*sfrp2)*, *p38 kinase*, *transforming growth factor β receptor IIa* (*tgfbr-iia*), *transcription factor protein*(*Gli*) a cytoplasmic polyadenylylation element (CPE), involved in Poly(A) tail elongation after the export of the mRNA to the cytoplasm, though a mechanism called cytoplasmic polyadenylation; in *Nemo-like kinase*, *Glycogen synthase kinase α*/*β*(*Gsk*) a polyadenylation response element *(*MOS-PRE); an AU found in *Smad4*, *Wnt ligand*, *p38 kinase*; in *Smad 4*, *Tgfbr-iib*, *Smoothened protein*, a GU-rich destabilization element, identified in human as a conserved sequence, involved in rapid mRNA turnover; in *Wnt signaling ligand*, *sfrp2*, *Gli*, there was a UNR binding site. The RNA binding protein UNR can act as an activator or inhibitor of translation initiation, promote mRNA turnover, or stabilizes mRNA.

### 2.7. ceRNA Network Reconstruction

**Figure 7:**
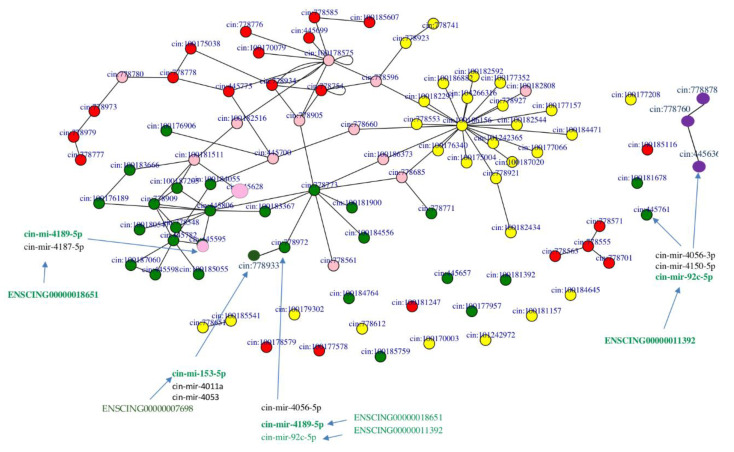
The four pathways are represented as networks formed by nodes connected by edges, and they are colored as follows: *Wnt*—green, *Tgf-β*—red and *FoxO*—yellow, *Hh*—purple. In RNA networks are also shown miRNAs and pseudogenes. Interacting ceRNAs are colored in green as they are interactors of Wnt pathway. The couple cin-mir-92c and ENSCING00000011392 is also ceRNA couple for HH2. Pink nodes represent proteins that are shared by two or more pathways.

**Figure 8:**
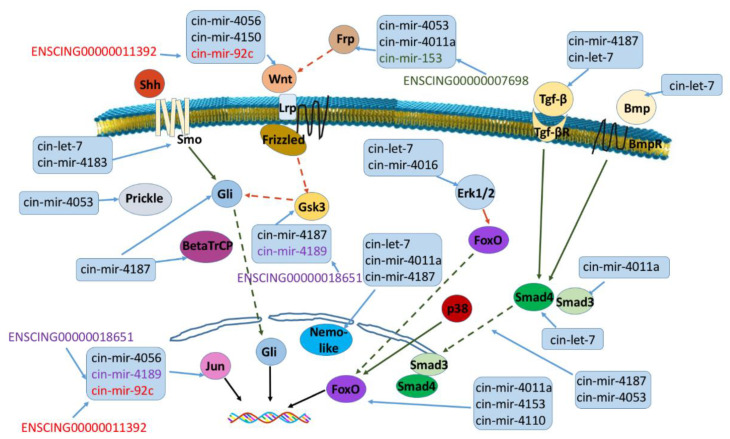
ceRNA network reconstruction of *Wn*t, *Tgf-β*, *FoxO* and *Hh* pathways. Schematic representation of RNA-interacting molecules evidenced by miRNATIP algorithm is shown. Red arrow is inhibition, green arrow is activation, dashed line in indirect link. The same color in a ceRNA interacting couple (miRNA and pseudogene) indicate a direct interaction between the two RNA molecules.

Gene enrichment and pathway analyses from NGS data, together with RNA-RNA interaction predictions through the miRNATIP predictor, evidenced a ceRNA interaction network involving four pathways linked to pharynx tissue homeostasis (Figure 7 and Figure 8) (Supplementary Material, network sheet). Table 5 shows a detailed list of these interactions. Indeed, different miRNAs interact with genes belonging to Tgf-β, Wnt, FoxO and Hh pathways, and they are both conserved and species-specific miRNAs. Conserved miRNAs are cin-let-7d-5p, cin-mir-153-5p, and cin-mir-92c-5p (Table 1). Moreover, three pseudogenes were shown to interact with miRNAs in these pathways: ENSCING00000011392, ENSCING00000018651, ENSCING00000007698 (Figure 7 and Figure 8). Ensembl annotation analysis showed that they could belong, respectively, to the annotation categories of *C. robusta* zinc finger protein (zf(c2h2)-31), Not4 protein (not4), and Rrp12-like protein (LOC100184722) (Table 4).

## 3. Discussion

In humans, numerous studies show the crucial role of transforming growth factor β (TGF-β) to regulate the self-renewal capacity of hematopoietic stem cells (HSCs) in vivo, precisely, in the bone marrow (BM) it maintains the homeostasis of normal hematopoiesis through the precise generation of mature blood cells [2,34,35,36,37,38]. Furthermore, the TGF-β signaling has an essential function to maintain the self-renewal capacity of HSCs, even when the tissue context changes [39,40].

In *Ciona robusta* the pharynx, its hematopoietic organ, consists of two epithelial monolayers aligned in transversal and longitudinal bars, where flows the haemolymph containing hemocytes, mainly lymphocyte-like cells, hyaline amoebocytes, morula cells, unilocular refractile granulocytes, signet ring cells [34]. In *C. robusta* following LPS challenge, Tgf-β cytokine was transcriptionally upregulated and expressed by the hemocytes that are found to flow inside the pharynx vessels. In upregulation of *Tgf-β* mRNA time profile (1–72 h) two phases can be recognized, the first (1–4 h) when the inflammatory response starts end the second (48–72 h) in which homeostasis is reached and maintained [30].

In this study, NGS and bioinformatics suggest that combinatorial and crosstalk at multiple levels, between TGF-β, WNT, FOXO, and Hedgehog (HH) signaling pathways are involved in pharynx tissue homeostasis.

The HH signaling pathway is evolutionarily conserved and is required for embryonic patterning, tissue repair, and regeneration. In vertebrates, HH signaling is controlled by polypeptide ligand, an intracellular signaling molecule called Hedgehog (HH). If the ligand is not present, two cell-surface transmembrane proteins called the patched receptor (PTCH1 or PTCH2) repress the 7-membrane-spanning receptor-like protein smoothened (SMO) leading to the phosphorylation of GLI (glioma-associated oncogene homolog) transcription factors by several protein kinases such as kinase A (PKA), GSK-3b, and CK1, and subsequent cleavage of GLI into truncated forms that act as repressors of HH target genes [41]. The binding of the HH ligand delete the inhibition of SMO leading to the activation and translocation of GLI proteins into the nucleus to control the expression of HH target genes [41].

In vertebrates, the WNT signaling pathways is involved in regulating many aspects of development and play important roles in cell-fate determination, self-renewal, and maintenance of stem cells [2]. Complete sequences of animal genomes have revealed that signaling pathways, such as TGF-β, WNT and FOXO [42,43,44,45] can be integrated to build an higher order networks and coordinated in signaling network that allows cells to read a limited set of cues and mount the diverse responses connected to the developmental processes and homeostasis [46].

In *C. robusta* gene enrichment and pathway analyses from NGS data, together with RNA–RNA interaction predictions evidenced a ceRNA (mRNA, miRNA and pseudogene) interaction network involved in post-transcriptional regulation of four pathways (Tgf-β, Wnt, FoxO, and Hh) linked to pharynx tissue homeostasis.

MicroRNAs (miRNAs), found in diverse eukaryotes from plants to animals, inhibit gene expression largely in a posttranscriptional manner, by interacting with miRNA response elements (MREs) in the mRNA 3′-UTRs of the target genes. Pseudogenes which have the similar MREs target genes of miRNAs [47], leading to direct competition for miRNAs and allowing pseudogenes to modulate gene expression [48,49], participating in the physiological maintenance of endogenous homeostasis and the pathological process of disease [50]. In *C. robusta* NGS identified 15,074 transcripts and of these 1% (201 elements) was classified as noncoding RNA (ncRNA). Of these 201, 90 were miRNAs and 27 pseudogenes. Gene enrichment and pathway analyses from NGS data, together with RNA–RNA interaction predictions by miRNATIP predictor, evidenced a ceRNA interaction network involving the four pathways linked to pharynx tissue homeostasis. Both conserved (cin-let-7d-5p, cin-mir-153-5p, and cin-mir-92c-5p) and species-specific miRNAs were found in ceRNA interaction network. Moreover, only three pseudogenes were found to interact with miRNAs involved in gene expression regulation of *Wnt* and *Hh* pathways.

After transcription, mRNAs are exported to the cytoplasm [51] to be translated. In the cytoplasm mRNAs can be turned over through regulated decay [52] by post-transcriptional regulatory steps important for gene expression. Studies show that interaction between RNA-binding proteins (RBPs) and specific cis-regulatory elements in target transcripts in 3′-UTR is the basis for post-transcriptional regulation of gene expression [53]. RegRNA analyses of the 3′-UTR mRNA of genes involved in *C. robusta* Tgf-β, Wnt, FoxO, and Hh signaling pathways showed a variety of cis-regulatory elements, including the CPE, ARE, MBE, GU-rich destabilization element, UNR binding site (an important cis-element for RNA regulation) and GAIT elements that, in humans, are implicated in post transcriptional regulation of several immune-related mRNAs, and have an important role in gene specific translation control in innate immunity [54].

All these data suggest that in *C. robusta*, as in humans, Tgf-β, FoxO, Wnt, and Hh signaling extensively cross-talk at many levels, and that multiple signaling inputs are integrated into the core transcription factor network to regulate target gene expression cooperatively in tissue homeostasis and the genes of these signaling pathways are subjected to a complex system of post-transcriptional regulations that involve in an integrated way miRNAs, pseudogenes and 3′UTR elements.

## 4. Materials and Methods

### 4.1. Tunicates

In this study, the model organism *Ciona robusta*, was formerly classified as *Ciona intestinalis.* Molecular studies have confirmed that *C. intestinalis* constitutes a compilation of species rather than a single species [55,56,57,58].

*C. robusta* specimens were collected from Sciacca harbor (Sicily, Italy) and were acclimatized and maintained as reported in Arizza et al. [33]

Fragments of pharynx tissue (200 mg) were immediately soaked in RNAlater tissue collection solution (AMBION, Austin, TX, USA) and Total RNA extraction was performed as reported in in Arizza et al. [33].

### 4.2. RNA Sequencing (RNA-Seq)

The RNA purity and quality of total RNA extracted from the pharynx of *C. robusta* (N = 3) were examined by NanoDrop and Agilent RNA 6000 Nano kits on an Agilent 2100 Bioanalyzer (AGILENT, Santa Clara, CA, USA), respectively. High-quality RNA samples (A260/A280 = 1.9–2.1, RIN ≥ 7) were used for cDNA library construction. RNA sequencing (RNA-Seq) was performed by BMR Genomics (Padua, Italy) on an Illumina platform in a single-end format 75 bp (1 × 75 bp) containing ~40 million ± 10% of reads/sample [33].

### 4.3. Bioinformatics Data Analysis

#### 4.3.1. Transcipts Analysis

All transcripts produced by NGS were annotated by Ensembl database (https://www.ensembl.org/index.html, release August 2020). The annotation evidenced different classes of RNA molecules: protein-coding and noncoding RNA. Between the ncRNAs the annotation evidenced the following RNA classes: miRNAs, pseudogenes, small nuclear RNA (snRNAs), small nucleolar RNAs (snoRNAs), ribosomal RNAs (rRNAs), miscellaneous RNAs (miscRNAs), and mitochondrial RNAs (mtRNAs).

#### 4.3.2. Gene Functional Enrichment

Gene Ontology enrichment analysis: *C.robusta* transcripts produced by NGS were analyzed through The Clusters of Orthologous Genes (COG) database (geneontology.org, release February 2021). All the three different Gene Ontology subcategories were investigated: (i) molecular functions (MF); (ii) cellular components (CC); (iii) and biological pathways (BP). The results for the three GO categories were filtered for *p*-value and for adjusted *p*-value (<0.05), and for Benjamini correction test (Benjamini < 0.05).

The Protein Analysis Through Evolutionary Relationships (PANTHER GO-slim analysis tool) system connected to the COG database was used to expand the annotation data of the gene and protein families obtained from GO. The “GO-slim” analysis mode was set, as it is more reliable and accurate than the GO annotation mode “GO-complete”.

#### 4.3.3. Analysis of Not-Annotated miRNAs

The sequences of the 36 miRNA precursors were aligned using miRBase Blastn alignment tool (mirbase.org, release October 2018). This tool allows us to find similar protein or nucleotide sequences to the target sequence under investigation. Each sequence was taken from the Ensembl genome browser (release 101, August 2020). Only “query” sequences with E-value < 0.05 and S score > 100 were considered as significant results.

E-value is defined as the probability due to chance, that there is another alignment with a similarity greater than the given S score. The S score is a measure of the similarity of the query to the sequence shown. As the typical threshold for a good E-value from a Blast search is e − 5 = (10 − 5) or lower, just a few sequences were considered as potential good results.

Sequence alignment was performed both with miRNA precursor sequences than with mature sequences. This choice allowed us to compare E-values results for each alignment to increase the power of analysis (sequence alignment with precursor and mature miRNAs are shown in supplementary files, miRBase blast mature and precursor sheets). Search sequence was done selecting stem-loop sequence, and miRNA mature; Blastn tool was applied as alignment, and E-value cutoff of 10 and species filter on *C. robusta* were selected to blast the “query” sequence.

#### 4.3.4. Analysis of Conserved miRNAs

To study evolution pattern of conserved *C. robu*sta homologues microRNA in animal genome: ZooMir (insr.ibms.sinica.edu.tw/ZooMir/index.php release April 2019) and MirGeneDB (https://mirgenedb.org/, release 2015) database of homologous microRNA genes search in animal genomes that have been validated and annotated. Results were also compared with miRNAs annotated in miRBase database (http://www.mirbase.org/ release October 2018).

#### 4.3.5. Analysis of Pseudogenes

The pseudogenes annotated by Ensembl database were analyzed through Basic Local Alignment Search Tool (Blastn tool) (blast.ncbi.nlm.nih.gov/Blast.cgi, release October 2020), to find regions of similarity between biological sequences and to see if pseudogenes produced by NGS derived by known genes and if they were conserved through evolution. The pseudogenes were analyzed according to Ensembl “genebuild” algorithm. Two parameters were used to evaluate the significance of the match: the expected value (E), a parameter that describes the number of hits one can “expect” to see by chance when searching a database of a particular size; and the score S (S), that is inversely correlated with the E value.

#### 4.3.6. RNA–RNA Interaction Prediction

RNA–RNA interaction predictions (i.e., miRNA–pseudogene and miRNA–mRNA target) were performed through the miRNA target interaction predictor (miRNATIP) algorithm [59]. For each RNA pair, miRNATIP exploits an approach that combines the advantages of an artificial neural network and the effectiveness of the relative binding free energy computation for each putative interaction.

The first step of the algorithm is the training of a self-organizing map (SOM) neural network [60]. This network is able to map high-dimensional datasets into a smaller dimensional space. The structure of the SOM is typically made by a two-dimensional lattice of interconnected neurons. By using competitive learning, all the sequences corresponding to miRNA seeds will be projected into this lattice (map), and all of them will be clustered according to their structural similarity. In other words, each miRNA seed will be as close to other miRNA seeds as they are similar, whereas different miRNA seeds will be as distant as their sequence are dissimilar. As a result of this phase, the map can be divided into areas where the input patterns share structural feature values. Each area represents a cluster. The second step deals with the projection of mRNA/pseudogene fragments on the trained map, in order to check if they can match a cluster. If fragments are compatible with a cluster, they will be considered for the next step. As a result, for each cluster, a list of miRNA seed-mRNA fragments is obtained, representing a preliminary list of putative interactions.

The third step, take care of clustered elements. For each pair of miRNA seed and mRNA/pseudogene fragment, miRNATIP compute the dissimilarity between the miRNA tail and an extended version of mRNA/pseudogene fragment (i.e., will be considered some nucleotides near to the fragment). The algorithm also considers potential bulges between miRNA seed and tail. If the computed dissimilarity is under a specific threshold, the putative interaction is considered for the next step. The last step filters out wrongly predicted interactions, by means of the computation of the binding free energy.

#### 4.3.7. Analysis of 3′UTR mRNA Target

To characterize the 3′UTR elements of mRNAs, a computational analysis was performed using the regulatory RNA motifs and elements finder tool (http://regrna.mbc.nctu.edu.tw/html/prediction.html, release February 2021).

#### 4.3.8. Pathway Enrichment Analysis

Pathway analysis was performed using the DAVID tool. First, functional annotation analysis was selected. The *C. robusta* gene list was compared with the reference *C. robusta* list present by the tool as background. Pathway annotation chart report was then selected, through the Kyoto Encyclopedia of Genes and Genomes (KEGG) pathway chart. DAVID pathway viewer displays user genes on static pathway maps generated by BioCarta and KEGG.

EASE (Exact Annotation Significance Enrichment) score threshold, a modified Fisher exact *p*-value used for functional annotation analysis, was set as 0.1. Results were considered statistically significant if *p*-value and adjusted *p*-value were < 0.05. The updated version of DAVID tool was 20 August 2020, DAVID release 6.7.

All statistical assessments of GO term enrichment and pathway analyses were performed by Fisher’s exact test in combination with a robust Benjamini correction test, for multiple testing. The row *p*-value and Benjamini thresholds were set as <0.05.

#### 4.3.9. ceRNA Network Construction and Visualization

To build ceRNA network evidenced by RNA-RNA interaction prediction, *C. robusta* pathways (Wnt, FoxO, Hh and Tgf-β) were downloaded from KEGG database (https://www.genome.jp/kegg/, release January 2021) in the Cytoscape tool (cytoscape.org, release March 2017). Cytoscape is an open-source software platform for visualizing molecular interaction networks and biological pathways and integrating these networks with annotations. The Cytokegg app was used to load KGML files, previously downloaded from KEGG, in Cytoscape db, and the STRING app was then used to convert the networks into STRING networks (STRINfy networks).

R package and R studio were then used to merge all pathways and to evidence common proteins (https://www.r-project.org/, release October 2020); (https://rstudio.com/products/rstudio/, release October 2020).

In particular, “igraph” package of R was used to analyses networks produced by STRING and to generate the image of integrated networks

## Figures and Tables

**Table 1:** MicroRNAs (miRNAs) conserved.

Transcript ID	Transcript Name
ENSCINT00000030081	cin-let-7a-2
ENSCINT00000030062	cin-let-7b
ENSCINT00000030060	cin-let-7c
ENSCINT00000030036	cin-let-7d
ENSCINT00000034599	cin-let-7f
ENSCINT00000030035	cin-mir-126
ENSCINT00000030087	cin-mir-141
ENSCINT00000030090	cin-mir-153
ENSCINT00000035589	cin-mir-375
ENSCINT00000030066	cin-mir-92°
ENSCINT00000030082	cin-mir-92c

**Table 2:** Species-specific miRNAs.

Transcript ID	Transcript Name
ENSCINT00000030078	cin-mir-1473
ENSCINT00000035831	cin-mir-3598
ENSCINT00000033430	cin-mir-4011
ENSCINT00000031162	cin-mir-4012-1
ENSCINT00000034078	cin-mir-4016-1
ENSCINT00000032003	cin-mir-4019
ENSCINT00000035935	cin-mir-4024
ENSCINT00000032443	cin-mir-4030
ENSCINT00000030503	cin-mir-4031
ENSCINT00000030983	cin-mir-4034
ENSCINT00000034251	cin-mir-4049
ENSCINT00000034742	cin-mir-4052
ENSCINT00000033641	cin-mir-4053
ENSCINT00000035897	cin-mir-4056
ENSCINT00000034395	cin-mir-4061
ENSCINT00000030710	cin-mir-4062
ENSCINT00000030744	cin-mir-4066
ENSCINT00000036059	cin-mir-4069
ENSCINT00000034132	cin-mir-4075
ENSCINT00000035254	cin-mir-4079
ENSCINT00000030637	cin-mir-4089
ENSCINT00000030429	cin-mir-4094
ENSCINT00000034249	cin-mir-4098
ENSCINT00000035687	cin-mir-4109
ENSCINT00000032059	cin-mir-4110
ENSCINT00000035827	cin-mir-4127
ENSCINT00000035006	cin-mir-4144
ENSCINT00000034925	cin-mir-4150
ENSCINT00000034736	cin-mir-4158
ENSCINT00000031184	cin-mir-4163
ENSCINT00000035929	cin-mir-4180
ENSCINT00000030226	cin-mir-4183
ENSCINT00000032396	cin-mir-4186
ENSCINT00000034165	cin-mir-4187
ENSCINT00000035972	cin-mir-4189
ENSCINT00000030905	cin-mir-4197
ENSCINT00000032545	cin-mir-4200
ENSCINT00000032095	cin-mir-5596b
ENSCINT00000037127	cin-mir-5598
ENSCINT00000030505	cin-mir-5600
ENSCINT00000031547	cin-mir-5605
ENSCINT00000035882	cin-mir-5609
ENSCINT00000033499	cin-mir-5611

**Table 3:** Evolution pattern of conserved miRNAs.

*C. elegans*	*D. melanogaster*	*S. purpuratus*	*B. floridae*	*C. robusta*	*D. rerio*	*X. tropicalis*	*G. gallus*	*M. musculus*	*H. sapiens*
mir-92	let-7	let-7	let7-a1	let7-a1	let7-a1	let-7a	let7-a1	let7-a1	let7-a1
mir-375	mir-92a	mir-92a	let-7a2	let-7a2	let-7a2	let7-b	let-7a2	let-7a2	let-7a2
	mir-92b	mir-92b	mir-92a1	let7-b	let-7a3	let-7c	let-7a3	let7-b	let-7a3
	mir-375	mir-92c	mir-92a2	let-7c	let-7a4	let-7e1	let7-b	let-7c1	let7-b
		mir-375	mir-92a3	let-7d	let-7a5	let-7e2	let-7c	let-7c2	let-7c
			mir-92b	let-7f	let-7a6	let-7f	let-7d	let-7d	let-7d
			mir-92c	mir-92a	let7-b	let-7g	let-7f	let-7e	let-7e
			mir-92d	mir-92b	let-7c1	let-7i	let-7g	et-7f1	et-7f1
			mir-153	mir-92c	let-7c2	mir-92a1	let-7i	let-7f2	let-7f2
			mir-375	mir-92d	let-7d1	mir-92a2	let-7k	let-7g	let-7g
				mir-92e	let-7d2	mir-92b	mir-92-1	let-7i	let-7i
				mir-126	let-7e	mir-126	mir-92-2	mir-92a1	mir-92a1
				mir-141	let-7f	mir-153-1	mir-126	mir-92a2	mir-92a2
				mir-153	let-7g1	mir-153-2	mir-153	mir-92b	mir-92b
				mir-375	let-7g2	mir-375	mir-375	mir-126	mir-126
					let-7i			mir-141	mir-141
					let-7j			mir-153	mir-153-1
					let-7k			mir-375	mir-153-2
					mir-92a1				mir-375
					mir-92a2				
					mir-92b				
					mir-126a				
					mir-126b				
					mir-141				
					mir-153a				
					mir-153b				
					mir-153c				
					mir-375-1				
					mir-375-2				

**Table 4:** Pseudogene identified by next-generation sequencing (NGS) and annotated by Ensembl database.

Pseudogene Gene ID	Chromosome	Paralogue	Accession Number	Identity	Chromosome
ENSCING00000018826	Scaffold HT000550.1: 1831–2416	Ubiquitin-conjugating enzyme E2 E1 (LOC100185453)	M_002129409.5	94.53%	9
ENSCING00000015967	Scaffold HT000041.1: 63,841–64,434	E3 ubiquitin-protein ligase synoviolin A-like (LOC100182895)	XM_018814866.2	99.67%	14
ENSCING00000001348	Scaffold HT000084.1: 79,125–80,530	PiggyBac transposable element-derived protein 4-like (LOC113475031)	XM_026838431.1	99.74%	Unplaced Scaffold
ENSCING00000005443	Chromosome 1: 2,817,744–2,819,238	Katanin p60 ATPase-containing subunit A1-like (LOC100178737)	XM_009863895.3	99.67%	1
ENSCING00000024145	Chromosome 14: 4,460,332–4,461,829	E3 ubiquitin-protein ligase synoviolin A-like (LOC100182895)	XM_018814866.2	99.67%	14
ENSCING00000006910	Chromosome 9: 241,768–243,963	U3 small nucleolar RNA-associated protein 14 homolog A (LOC100182485)	XM_002119512.5	100.00%	9
ENSCING00000024624	Chromosome 1: 9,732,896–9,735,685	Apoptosis-stimulating of p53 protein 1 (LOC100181784)	XM_002123552.5	100.00%	1
ENSCING00000021320	Scaffold HT000124.1: 299,056–300,232	zinc finger protein (zf(c2h2)-32)	NM_001078404.1	98.98%	Unplaced Scaffold
ENSCING00000015544	Scaffold HT000124.1: 300,647–301,709	zinc finger protein (zf(c2h2)-32)	NM_001078404.1	95.29%	Unplaced Scaffold
ENSCING00000005867	Chromosome 2: 2,842,058–2,843,557	tRNA modification GTPase GTPBP3, mitochondrial (LOC100183076)	XM_002128700.5	98.20%	2
ENSCING00000011392	Chromosome 2: 5,498,086–5,500,091	zinc finger protein (zf(c2h2)-31)	NM_001078403.1	98.80%	2
ENSCING00000018962	Chromosome 4: 3,953,395–3,954,891	HSF protein (hsf)	NM_001078269.1	99.26%	4
ENSCING00000000148	Scaffold HT000121.1: 61,799–64,445	Toll-like receptor 1 (ci-tlr1)	NM_001166127.2	100.00%	Unplaced Scaffold
ENSCING00000008834	Chromosome 4: 4,639,472–4,640,938	FoxB protein (foxB)	NM_001032523.1	99.65%	4
ENSCING00000001425	Scaffold HT000145.1: 8,837–11,898	Phosphatidylinositol 4,5-bisphosphate 3-kinase catalytic subunit alpha isoform-like (LOC100180252)	XM_004227228.4	99.80%	Unplaced Scaffold
ENSCING00000007135	Chromosome 3: 220,836–221,952	Transcription factor protein (lag1-like3)	NM_001100125.1	99.37%	3
ENSCING00000019275	Chromosome 11: 158,872–160,388	28S ribosomal protein S34, mitochondrial-like (LOC100182579), transcript variant X1	XM_002128752.5	99.87%	11
ENSCING00000022279	Chromosome 3: 892,375–893,636	Aldehyde dehydrogenase, dimeric NADP-preferring-like (LOC100185488)	XM_009859599.3	99.76%	3
ENSCING00000019638	Chromosome 13: 1,743,172–1,744,608	poly [ADP-ribose] polymerase 2 (LOC100178364)	XM_002128271.4	100.00%	13
ENSCING00000009399	Chromosome 3: 3,711,873–3,713,223	major facilitator superfamily domain-containing protein 10-like (LOC100182178)	XM_009859732.3	99.85%	3
ENSCING00000006160	Scaffold HT000098.1: 302,094–303,016	Not4 protein (not4)	NM_001032434.1	99.67%	Unplaced Scaffold
ENSCING00000018651	Scaffold HT000098.1: 863,758–864,748	Uncharacterized LOC494370 (LOC494370), mRNA	NM_001245041.1	99.90%	Unplaced Scaffold
ENSCING00000019488	Chromosome 11: 4,823,586–4,824,678	p38 kinase (p38)	NM_001078490.1	99.18%	11
ENSCING00000008915	Chromosome 3: 5,548,986–5,553,157	DNA-directed RNA polymerase III subunit RPC1-like (LOC100185039)	XM_009859781.3	99.86%	3
ENSCING00000001594	Scaffold HT000119.1: 138,999–140,115	Betaine-homocysteine S-methyltransferase 1 pseudogene (LOC104266620)	XR_001975199.2	99.91%	Unplaced Scaffold
ENSCING00000007698	Chromosome 12: 2,614,369–2,618,285	RRP12-like protein (LOC100184722)	XM_009862261.3	99.92%	12
ENSCING00000018747	Chromosome 8: 4,581,662–4,582,653	zinc finger protein (zf(c2h2)-21)	NM_001078395.1	99.70%	8

**Table 5:** mRNA-miRNA-pseudogene interaction network of *Tgf-β*, *Wnt*, *FoxO*, and *Hh* pathways. miRNA and pseudogenes interacting couples are showed in the same row.

Pathway	Accession Number	Gene Name	miRNA	Pseudogenes
*Wnt*	NM_001078300	*Nemo-Like kinase(Nemo-like)*	*cin-let-7d-5p*, *cin-mir-4187-5p* *cin-mir-4011a-5p*	
	NM_001078476	*Smad4 protein(Smad4)*	*cin-let-7d-5p*	
	NM_001078326	*Wnt signaling ligand (LOC778720)*	*cin-mir-4056-3p* *cin-mir-4150-5p*	
			*cin-mir-92c-5p*	ENSCING00000011392
	NM_001032454	*beta-transducin repeat-containing homologue protein(BetaTrCP)*	*cin-mir-4187-5p*	
	NM_001032425	*glycogen synthase kinase alpha/beta(Gsk)*	*cin-mir-4189-5p*	ENSCING00000018651
			*cin-mir-4187-5p*	
	NM_001122968	*prickle 2(Pk2)*	*cin-mir-4053-5p*	
	NM_001078496	*secreted frizzled-related protein(Sfrp1/5)*	*cin-mir-153-5p*	ENSCING00000007698
	NM_001078536	*secreted frizzled-related protein(Sfrp2)*	*cin-mir-4011a-5p*, *cin-mir-4053-5p*	
	NM_001078528	*transcription factor protein(Jun)*	*cin-mir-4056-5p*	
			*cin-mir-4189-5p*	ENSCING00000018651
			*cin-mir-92c-5p*	ENSCING00000011392
*FoxO*	NM_001078300	*Nemo-Like kinase(Nemo-like)*	*cin-let-7d-5p*, *cin-mir-4187-5p*, *cin-mir-4011a-5p*	
	NM_001078349	*Smad2/3b protein(Smad2/3b)*	*cin-mir-4011a-5p*	
	NM_001078476	*Smad4 protein(Smad4)*	*cin-let-7d-5p*	
	NM_001078229	*mitogen-activated protein kinase(Erk1/2)*	*cin-let-7d-5p*, *cin-mir-4016*	
	NM_001078490	*p38 kinase(P38)*	*cin-mir-4187-5p*, *cin-mir-4053-5p*	
	NM_001078249	*transcription factor protein(Foxo)*	*cin-mir-4053-5p* *cin-mir-4150-5p* *cin-mir-4011a-5p*	
	NM_001078368	*transforming growth factor beta receptor(Tgfbr-iib)*	*cin-mir-4075-5p*, *cin-let-7d-5p*	
	NM_001078194	*uncharacterized LOC778553(LOC778553)*	*cin-mir-4187-5p*	
*Tgf-β*	NM_001078349	*Smad2/3b protein(Smad2/3b)*	*cin-mir-4011a-5p*	
	NM_001078476	*Smad4 protein(Smad4)*	*cin-let-7d-5p*	
	NM_001078229	*mitogen-activated protein kinase(Erk1/2)*	*cin-let-7d-5p*, *cin-mir-4016*	
	NM_001078367	*transforming growth factor beta receptor(Tgfbr-iia)*	*cin-mir-4187-5p*	
	NM_001078368	*transforming growth factor beta receptor(Tgfbr-iib)*	*cin-let-7d-5p*	
	NM_001078196	*transforming growth factor beta superfamily signaling ligand(Bmp5/7-like)*	*cin-let-7d-5p*	
*Hh*	NM_001032454	*beta-transducin repeat-containing homologue protein(BetaTrCP)*	*cin-mir-4187-5p*	
	NM_001032425	*glycogen synthase kinase alpha/beta(Gsk)*	*cin-mir-4189-5p*	ENSCING00000018651
			*cin-mir-4187-5p*	
	NM_001078351	*smoothened protein(Smoothened)*	*cin-let-7d-5p* *cin-mir-4183-5p*	
	NM_001078483	*transcription factor protein(Gli)*	*cin-mir-4187-5p*	

## Supplementary Materials

The following are available online at https://www.mdpi.com/article/10.3390/ijms22073497/s1.
