# Supplementary material for: ceRNA Network Regulation of TGF-β, WNT, FOXO, Hedgehog Pathways in the Pharynx of Ciona robusta
Source: Int J Mol Sci. 2021 Mar 28;22(7):3497. doi: 10.3390/ijms22073497 (PMC8037537; doi:10.3390/ijms22073497)
Supplement: Supplementary file 1 [file ijms-22-03497-s001.zip › ijms-1144418 suppl/supplementary materials.pdf]

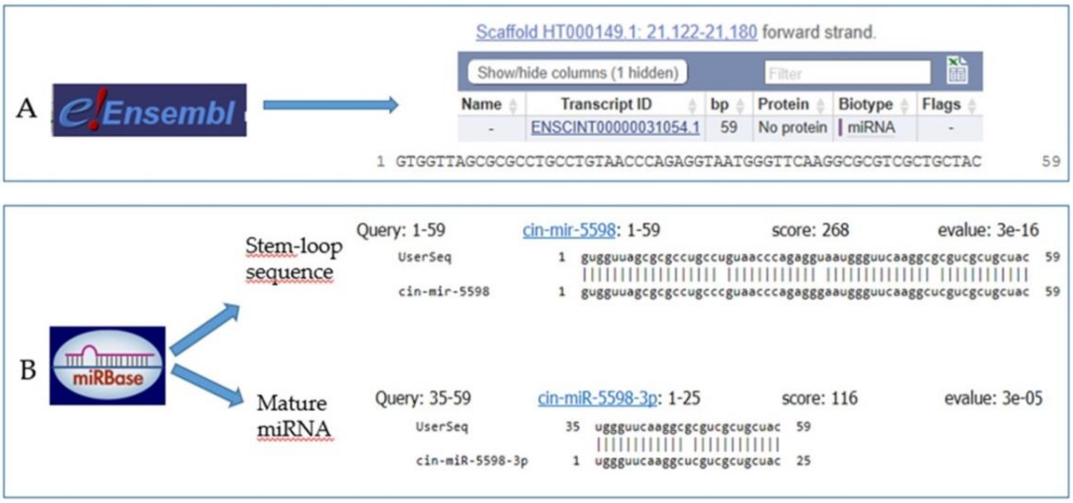

Figure S1: **T**he pipeline used to annotate unknown miRNAs. **A**. miRNA annotation by Ensembl databas. **B**. alignment of miRNA sequence using miRBase Blastn tool, both with stem-lop and with mature sequence.
